# Sharing Patient Technology Preferences With Dementia Care Networks: The Let’s Talk Tech Decision Aid

**DOI:** 10.1093/geroni/igaf122.1768

**Published:** 2025-12-31

**Authors:** Clara Berridge, Natalie Turner, William Lober, George Demiris, Jeffrey Kaye

**Affiliations:** University of Washington, Seattle, Washington, United States; University of Washington, Seattle, Washington, United States; University of Washington, Seattle, Washington, United States; University of Pennsylvania, Philadelphia, Pennsylvania, United States; Oregon Health & Science University, Portland, Oregon, United States

## Abstract

Let’s Talk Tech (LTT) is a self-administered web intervention for people with memory loss and their care partners that supports decision-making about digital health technologies. In past work, study participants wanted to share LTT technology preference reports with their larger care networks. This study aims to understand with whom care dyads want to share their technology preference reports and why, and if and how clinicians want to receive them. Together, fifteen dyads of people living with MCI or early-stage dementia (n = 15) and a care partner (n = 15) completed LTT and two survey questions. Care partners completed independent follow-up interviews, and 32 clinicians at four Alzheimer’s Disease Research Center-affiliated clinics viewed an LTT report and completed a 10-question survey. We used descriptive statistics for survey responses and thematic analysis for interviews. Two-thirds of care partners (n = 10) wanted to share the report with family members. Half (n = 8) wanted to share it with clinicians to keep them informed about the dyad’s planning and facilitate conversations about technology options. Clinicians were highly receptive to accessing technology preference reports in EHRs and to having discussions about technology be a part of advance care planning. Thirty of 32 clinicians reported they would want their patients’ technology preferences reports, with 25 wanting to access it via the EHR. Findings demonstrate potential value to both family members and providers of sharing technology preferences beyond the care dyad. Future research should test integration in the EHR and the potential of sharing technology preferences to support person-centered technology choices.

